# Prevalence, Clinical Features, and Management of Cervical Polyps: A One-Year Study at Rabia Balkhi Hospital in Afghanistan

**DOI:** 10.7759/cureus.69417

**Published:** 2024-09-14

**Authors:** Bibi Sarah Yousofzai, Khalida Walizada, Maliha Ahmadi, Mahjuba Ansari, Rana Beloulou Latoui, Maria Fatima, Rida Mehmood, Muhammad Subhan, Ruqiya Bibi, Seth Omari Mensah

**Affiliations:** 1 Obstetrics and Gynecology, Be Team International Cure Hospital, Kabul, AFG; 2 Neurological Surgery, Ali Abad Teaching Hospital, Kabul, AFG; 3 Obstetrics and Gynecology, Rabia Balkhi Hospital, Kabul, AFG; 4 Family Medicine, Be Team International Cure Hospital, Kabul, AFG; 5 Obstetrics and Gynecology, CHU Ibn Rochd Hospital, Annaba, DZA; 6 Internal Medicine/Family Medicine, Windsor University School of Medicine, Detroit, USA; 7 Medicine, Rawalpindi Medical University, Rawalpindi, PAK; 8 Internal Medicine, Allama Iqbal Medical College, Lahore, PAK; 9 Medicine, Allama Iqbal Medical College, Lahore, PAK; 10 Obstetrics and Gynecology, Royal Free Hospital, London, GBR

**Keywords:** blood loss anemia, cervical polyps, dilatation and curettage, hysterectomy, risk of malignancy

## Abstract

Background

Cervical polyps are benign growths from the cervical epithelium, found in 2% to 5% of the population. They usually appear as small, stalk-attached lesions ranging from pea to cherry size. Contributing factors include cervical infections, congestion, and elevated estrogen levels. During pregnancy, these polyps may increase the risk of late abortion and preterm birth. While malignancy is rare, histopathological examination after removal is advised to rule out cancer.

Materials and methods

This current study, the first of its kind in Afghanistan, aimed to assess the scope of cervical polyps among Afghan women presenting to Rabia Balkhi National Hospital in 2018. We conducted a descriptive study of cervical polyps, exploring the prevalence, symptoms, complications, and procedures used to remove them. Data were collected from 3730 cases admitted to the hospital.

Results

Out of 3730 cases, 50 patients (1.34%) were diagnosed with cervical polyps. The polyps varied in size and were typically attached to the cervix by a slender stalk. Symptoms reported included inter-menstrual, post-coital, and post-menopausal bleeding. Histopathological examinations were performed following polyp removal to rule out malignancy.

Conclusion

This study provides a comprehensive overview of the prevalence and clinical presentation of cervical polyps among Afghan women. The findings highlight the importance of recognizing and managing cervical polyps to prevent potential complications, especially during pregnancy. Further research is needed to understand this population's epidemiology, pathogenesis, and optimal management strategies for cervical polyps.

## Introduction

Cervical polyps are benign lesions caused by focal hyperplasia of the cervical epithelium and typically appear between 2% and 5% of women [1,2]. The size ranges from pea to cherry, and a slim stalk attaches most to their host cervix [3]. Cervical polyps are composed of vascular stroma that is susceptible to being affected by cervical infections, congestion, and elevated estrogen levels [3,4]. Their prevalence in both America and Europe ranges between 2% and 5%; they tend to occur more commonly among women aged 20+ who have had multiple pregnancies [4,5]. Risk factors for polyps include chronic cervical inflammation, sexually transmitted infections, and hormonal imbalances [4,5]. Polyps can cause intermenstrual, postcoital, and postmenopausal bleeding and increase the risk of late abortion and spontaneous preterm birth during gestation [5,6]. Although malignancy is relatively rare, histopathological examination should always be undertaken to detect and rule cancer cells out [6]. Cervical polyps are caused by focal hyperplasia of the columnar epithelium of the endocervix, with inflammation and abnormal estrogen responses being two potential contributing factors [6,7]. Histological testing confirms the diagnosis, with additional investigations including pelvic exams, cervical smears, and ultrasound scans to establish their presence [7]. Management usually entails polypectomy, an outpatient minor procedure with biopsy as an essential part of its monitoring for malignancy screening [7-10]. This study aims to assess the scope, pattern, and frequency of cervical polyps among Afghan women presenting to Rabia Balkhi National Hospital in 2018. Our research provides a descriptive analysis of the prevalence, symptoms, complications, and procedures conducted to remove these polyps.

## Materials and methods

This descriptive, cross-sectional study aimed to assess the prevalence, clinical presentation, and management of cervical polyps among patients admitted to Rabia Balkhi Hospital, Kabul, Afghanistan, in 2018. The primary focus was to identify patterns in the age distribution, presenting symptoms, and procedural outcomes associated with cervical polyps in the Afghan population.

Definition

Cervical polyps are benign growths arising from the hyperplasia of the cervical epithelium, as small, stalk-like growths on the cervix.

Study population

The study population consisted of women admitted to Rabia Balkhi Hospital in Kabul, Afghanistan, during the calendar year 2018. Out of 3730 total admissions, 50 women were diagnosed with cervical polyps, representing 1.34% of the admitted patient population. This group of 50 women formed the sample for this study.

Study participants

Inclusion criteria for study participants were all women diagnosed with cervical polyps during 2018. Women who had incomplete medical records or a diagnosis other than cervical polyps were excluded from the study. The participants included women of various age groups, reproductive histories, and presenting symptoms, providing a diverse representation of cervical polyp cases.

Data collection

Data were collected from patient medical records, hospital registers, ultrasound reports, and gynecological procedure logs, including Dilation and Curettage (D&C) protocols. Information gathered included patient demographics (age, parity), clinical presentation (e.g., intermenstrual bleeding, post-coital bleeding), and procedural outcomes. Histopathological examination reports were reviewed to confirm the diagnosis and assess the potential for malignancy. Additionally, a questionnaire was employed to supplement missing data through patient interviews when necessary.

Data analysis

The data were entered and analyzed using Microsoft Excel. Descriptive statistics such as means, frequencies, and percentages were used to summarize the data. The chi-square test was used for categorical variables, and t-tests were employed for continuous variables. A p-value of less than 0.05 was considered statistically significant. The analysis focused on identifying age-based trends, presenting symptoms, and procedural outcomes, comparing these with global data from published studies. Ethical approval was granted by the Ministry of Public Health of Afghanistan. Consent was waived due to the de-identified nature of the data, as approved by the Institutional Review Board (IRB) of the Afghanistan Public Health Institute. Data collection was authorized by the Medical Records Department of Cure International Hospital in Kabul. The study complied with the ethical principles of the Declaration of Helsinki.

## Results

General gynecological consultations in 2018

In 2018, 20,844 patients were seen for general gynecological consultations at Rabia Balkhi Hospital. Among them, 3,730 (18%) were inpatients, and 17,114 (82%) were outpatients.

Incidence of cervical polyps and management

Among the 3,730 gynecology inpatients, 50 cases of cervical polyps were identified, accounting for 1% of all gynecological admissions. Of these, 10% (n=5) underwent hysterectomy as part of their management, while 90% (n=45) were managed through non-surgical approaches.

Clinical characteristics of cervical polyp patients

Most cervical polyp cases were identified in women over 35 years of age (56%), while 40% of cases presented vaginal bleeding as the most common symptom, followed by bleeding during intercourse (26%) and pain during intercourse (20%). Additionally, anemia was prevalent in these patients, with 48% having hemoglobin levels between 7 and 9 g/dL. Only 22% of patients required a blood transfusion.

Histopathological examination

After surgical procedures, samples were sent for histopathological examination. It was found that approximately 2% of cervical polyps were malignant. This highlights the importance of histopathological evaluation to accurately determine the malignancy potential of cervical polyps.

All the important data are consolidated in Table 1 below to streamline the results.

**Table 1:** Summary of cervical polyp cases and key findings

Category	Number of patients	Percentage (%)
Total gynecology inpatients	3,730	100%
Cervical polyp cases	50	1%
Non-cervical polyp cases	3,680	99%
Hysterectomy for cervical polyps	5	10%
Non-hysterectomy cases	45	90%
Age > 35 years	28	56%
Vaginal bleeding	20	40%
Bleeding during intercourse	13	26%
Pain during intercourse	10	20%
Anemia (Hb 7-9 g/dL)	23	48%
Blood transfusion required	11	22%
Malignant polyps (histopathology)	1	2%

## Discussion

Cervical polyps are uncommon, with an incidence ranging from 2% to 5% in the general population [1]. While most polyps are benign, approximately 0.2% to 1.5% of cervical polyps (2 to 15 cases per 1,000) can undergo malignant transformation [1]. Additionally, it is noteworthy that between 7.8% and 50% of women (approximately 78 to 500 per 1,000 women) may develop polyps in the lower reproductive tract at some point in their lives [7]. These lesions originate from the ecto-cervical or endo-cervical canal of the uterus, with ecto-cervical polyps predominantly affecting postmenopausal women. In contrast, endo-cervical polyps are more common in premenopausal women [1,2].

Cervical polyps are often asymptomatic in about two-thirds of women, which means approximately 66% of women with cervical polyps do not experience symptoms [3,4]. Still, when symptoms do occur, they may include intermenstrual bleeding, postcoital bleeding, postmenopausal bleeding, hypermenorrhea, and infertility [1-3,7]. The potential complications during pregnancy, such as late abortion and an increased risk of spontaneous preterm birth, underscore the clinical significance of these lesions, particularly due to cervical insufficiency in early pregnancy [4]. Although the malignant potential of cervical polyps is low, particularly in the premenopausal period, the risk increases during peri-menopausal and post-menopausal phases, warranting careful monitoring and histopathological evaluation post-excision [8]. Our study, conducted at Rabia Balkhi National Hospital in 2018, identified cervical polyps in 1% of the total admitted cases, with 50 out of 3,730 admissions of women patients. The majority of affected patients were over 35 years old (56%, N=28) and had high parity, with 70% (N=35) having a gravidity of more than five pregnancies. Vaginal bleeding was the most prevalent clinical presentation, observed in 40% of cases (N=20), often leading to iron deficiency anemia. Notably, 48% (N=11) of anemic patients had hemoglobin levels between 7 g/dL and 9 g/dL, and 22% (N=11) required blood transfusions. These findings highlight the importance of identifying and managing cervical polyps in clinical practice.

Our data also revealed that 90% (N=45) of cases underwent dilatation and curettage (D&C), with the remaining 10% (N=5) requiring hysterectomy. These findings are consistent with the recommendations in the literature, which suggest that polyps larger than 10 mm or those causing significant symptoms should be surgically removed and subjected to histopathological examination to rule out malignancy [7]. In contrast, smaller polyps may be managed conservatively with regular monitoring [7].

Several studies suggest that factors such as cervical congestion, infection, inflammation, and elevated estrogen levels may contribute to the development of cervical polyps [1]. A study conducted at the University of Illinois described these polyps as benign lesions arising from hyperplasia of the endo-cervical canal, characterized by a vascular stroma covered by either squamous or columnar epithelium [2]. Given the variability in the size of these polyps, which can range from millimeters to up to 3 cm, the clinical approach to management must be tailored to the individual patient [2].

Studies from other regions have provided valuable insights into the characteristics and management of cervical polyps. For instance, the Department of Health Care in India conducted a descriptive analysis in 2016, finding that the highest incidence of cervical polyps occurred in women aged 40-49 years [9]. Similarly, a study from Pakistan reported a low malignancy rate (0.1%, N=4) among 4,063 cases of cervical polyps but emphasized the importance of resection and histopathological examination to identify potential dysplastic or metaplastic changes [10].

Our study is among the first to examine the incidence and management of cervical polyps in Afghanistan. However, as it was conducted in a single hospital, the findings may not be generalizable to the broader population. Future studies with larger, multi-center cohorts are needed to fully understand the epidemiology and clinical outcomes of cervical polyps in this region.

Limitations

This study has several limitations. It was conducted at a single center; its findings may not be generalizable to other settings. The retrospective design depends on the accuracy of existing medical records, which may be incomplete or biased. Without a control group, comparative data is lacking, and the study’s focus on demographic and clinical characteristics does not explore potential risk factors. Additionally, the research was limited to a single year, potentially missing variations over time or seasonal effects.

## Conclusions

This study emphasizes the proactive management of cervical polyps through their removal and detailed histological examination to assess malignancy potential. Pre-polypectomy cervical smear cytology is crucial for early detection of malignancies, aiding in tailored treatment plans. Additionally, endometrial sampling is recommended, especially for postmenopausal women, to ensure comprehensive reproductive health assessment. Addressing cervical polyps promptly can prevent symptoms like abnormal bleeding and complications such as anemia, ultimately improving patient outcomes. A thorough approach from initial evaluation to ongoing monitoring is essential for maintaining reproductive health and reducing risks associated with untreated cervical polyps.
